# OCD symptoms in succinic semialdehyde dehydrogenase (SSADH) deficiency: a case report

**DOI:** 10.1186/s12888-020-02794-8

**Published:** 2020-08-05

**Authors:** Sachin Phakey, Thomas Rego, Frank Gaillard, Julie Panetta, Andrew Evans, Gerard De Jong, Mark Walterfang

**Affiliations:** 1 Neuropsychiatry Unit, Royal Melbourne Hospital, Grattan St, Parkville, VIC 3050 Australia; 2 Faculty of Medicine, Dentistry and Health Sciences at The University of Melbourne, Grattan St, Parkville, VIC 3010 Australia; 3 Department of Psychiatry, University of Melbourne, Parkville, VIC 3010 Australia; 4 Department of Radiology, Royal Melbourne Hospital, Grattan St, Parkville, VIC 3050 Australia; 5 Metabolic Diseases Unit, Royal Melbourne Hospital, Grattan St, Parkville, VIC 3050 Australia; 6 Department of Neurology, Royal Melbourne Hospital, Grattan St, Parkville, VIC 3050 Australia; 7 Melbourne Neuropsychiatry Centre, University of Melbourne, Parkville, VIC 3010 Australia

**Keywords:** SSADH deficiency, Neuropsychiatry, Metabolic disorders, Obsessive-compulsive, Case report

## Abstract

**Background:**

Succinic semialdehyde dehydrogenase (SSADH) deficiency is a rare neurometabolic disorder resulting in a heterogeneous clinical phenotype. Adolescent and adult patients with SSADH deficiency may present with OCD symptoms. There is minimal literature regarding the pathological basis of OCD symptoms and their management amongst SSADH deficiency patients.

**Case presentation:**

A 26-year-old woman with SSADH deficiency experienced obsessional slowness and hesitancy in her activities of daily living, with motor rituals and stereotypies of her hands and face. Neuroimaging revealed T2 hyperintensities of the globi pallidi bilaterally. Commencement of the serotonergic escitalopram moderately improved her OCD symptoms. The addition of the dopaminergic pramipexole hydrochloride yielded further improvement, following unsuccessful trial of other adjuncts: risperidone, methylphenidate and mirtazapine.

**Conclusions:**

Pallidal pathology may explain the manifestation of OCD symptoms amongst individuals with SSADH deficiency. Serotonergic and concomitant dopaminergic therapy may be a viable treatment regimen for SSADH deficiency patients presenting with OCD symptoms.

## Background

Succinic semialdehyde dehydrogenase (SSADH) deficiency (OMIM #271980), also known as 4-hydroxybutyric aciduria, is an autosomal recessive disorder in the degradation pathway of γ-aminobutyric acid (GABA): the central nervous system’s predominant inhibitory neurotransmitter [1–3]. In SSADH deficiency, there is an accumulation of the compound γ-hydroxybutyric acid (GHB) (4-hydroxybutyric acid) within the cerebrospinal fluid, serum and urine [1, 4]. Urinary organic acid analysis is used as a means of detecting GHB and diagnosing SSADH [5]. Neuroimaging is often useful as part of the diagnostic work-up. Magnetic resonance imaging (MRI) frequently reveals T2-weighted hyperintensities involving the globi pallidi, subcortical white matter, cerebellar dentate nuclei and brainstem [5, 6]. A normal MRI may be observed in up to 43% of cases [6]. Confirmation of diagnosis may be achieved by genetic analysis of the *ALDH5A1* gene encoding SSADH.

SSADH deficiency is rare, identified in approximately 450 patients worldwide [7]. SSADH deficiency has non-specific symptomatology and a varied natural history throughout the life course [2, 5, 6, 8–10]. Hence, SSADH deficiency is likely underdiagnosed [7, 9, 10]. In infancy and childhood, individuals with SSADH deficiency typically present with global developmental delay, hypotonia, epilepsy and ataxia [5, 8, 9]. In addition to these clinical features, sleep disorders and neuropsychiatric symptoms are common manifestations in adolescence and adulthood [6, 7, 9, 10]. Obsessive-compulsive disorder (OCD) symptoms have previously been observed in adolescents and adults [7, 9, 10]. Management of SSADH deficiency patients remains largely symptomatic [7, 11]. Management of OCD symptoms in SSADH deficiency patients remains unclear and is generally guided by conventional guidelines for OCD treatment.

We describe the case of a 26-year-old woman with SSADH deficiency who developed obsessive-compulsive disorder (OCD)-like symptoms and our subsequent management approach. This case provides a possible framework for the management of future SSADH patients who present with similar symptoms.

## Case presentation

Ms. A is a 26-year-old single unemployed woman referred to a tertiary Neuropsychiatry Unit for ongoing management of neuropsychiatric symptoms of SSADH deficiency. Delayed developmental milestones and seizures were noted from an early age. At 4 years of age, Ms. A was diagnosed with epilepsy and global developmental delay of unknown aetiology. No further work-up was conducted. She was treated with antiepileptic medication and early psychosocial intervention, attending specialised primary and secondary schools.

At 12 years of age, Ms. A was diagnosed with SSADH deficiency following the diagnosis of her younger brother with the same condition. Her diagnosis was confirmed via urinary organic acid quantification, with elevated 4-hydroxybutyric acid and 4,5-dihydroxy hexanoic lactone levels. Genotyping was not performed. Formal neuropsychological testing at the time revealed mild-to-moderate intellectual disability.

From age 14, Ms. A experienced significant functional decline with manifestation of worsening OCD symptoms. She displayed marked difficulties initiating tasks, obsessional slowness and hesitancy in her activities of daily living, particularly when toileting and eating resulting in incontinence and weight loss, driven by obsessional doubt (about whether she was eating correctly or too quickly, had lifted her leg the correct way to put on trousers), and fears that certain behaviours may result in harm to her mother or brother. She exhibited motor rituals, particularly regarding the placement of food utensils and the correct “cutting” of food, and stereotypies of her hands and face involving wiping and chewing. Mealtimes were particularly problematic: prefaced by 30 min of pacing, 30–45 min of ordering cutlery, and then 1–2 h of slow mastication, marked by frequent pauses, requiring prompting to continue. She denied intrusive internal imagery. There were no religious or sexual preoccupations, counting or cleaning behaviours. Delusions and hallucinations were not apparent. Ms. A also showed increasing agitation and anxiety. When 22-years-old, Ms. A was referred to the Neuropsychiatry Unit for assessment and symptomatic management of these symptoms. Her score on the Yale-Brown Obsessive-Compulsive Scale (Y-BOCS) at initial assessment was 33, denoting extreme OCD (0–13 mild, 14–25 moderate, 26–34 moderate-severe OCD; 35–40 severe OCD) [12]. On mental state, she was well-groomed and politely-engaged. She had markedly reduced eye contact and a mild fine tremor of her upper limbs. Her speech was slow, had low tone and was mildly dystonic. Her vocalisations were slow, with long latency and were brief. Her affect was restricted yet congruent with her mood. There were no psychotic phenomena, yet she was preoccupied with internal cognitions of doubt and uncertainty. Neurological examination revealed mildly elevated tone, particularly on augmentation, with cogwheeling and hyperreflexia bilaterally. She demonstrated occasional orobuccal lip-sucking movements and athetoid movements of the trunk. She had reduced arm swing when walking, pedestal turning, with reduced foot clearance and stride length. Eye movements showed some saccadic intrusions into slow pursuit movements, and saccadic over-and under-shoot requiring multiple small corrections. Her only treatment at this time was lamotrigine 75 mg bd.

**Fig. 1:**
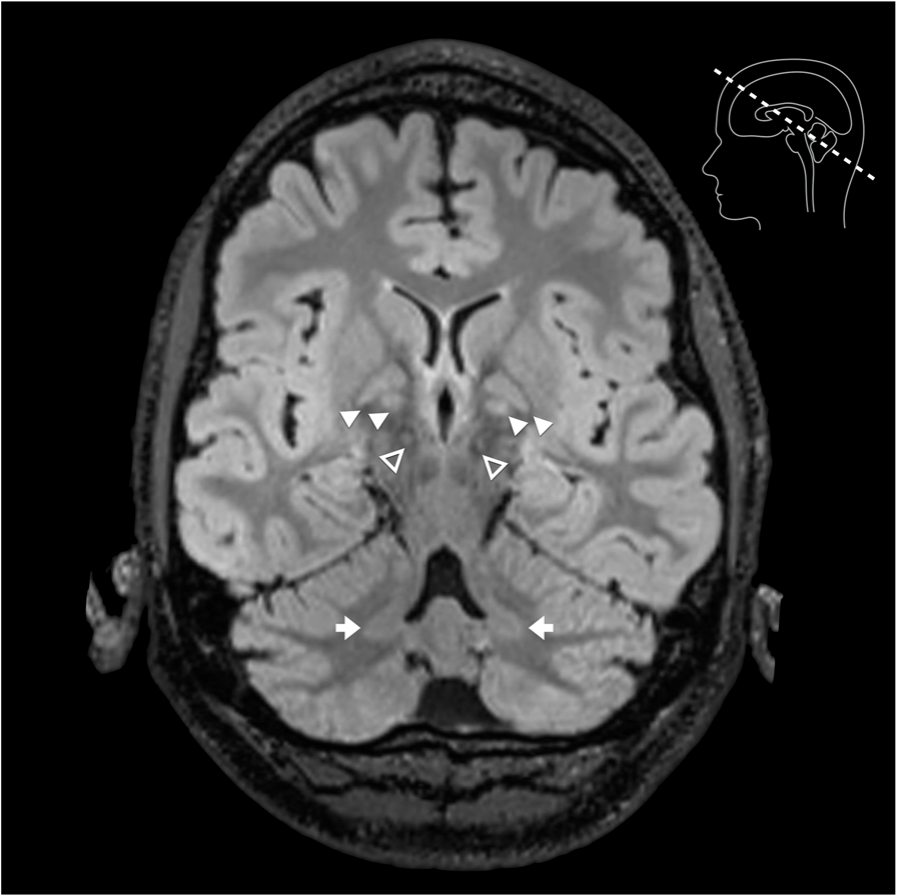
Oblique axial FLAIR image demonstrating increased T2 signal in the pars interna and externa of the globi pallidi (solid arrow heads), substantiae nigrae (open arrow heads) and dentate nuclei (arrows)

Neuroimaging revealed significant increased T2 signal in the pars interna and externa of the globi pallidi, substantiae nigrae and dentate nuclei bilaterally (Fig. 1). Moderate volume loss was also evident involving primarily the frontal lobe white matter, deep grey matter nuclei, brainstem and cerebellum.

Ms. A commenced the serotonergic medication escitalopram, which was gradually up-titrated from 10 mg to 70 mg od. This moderately improved her obsessional slowness, psychomotor retardation and motor rituals. Spontaneity in initiating movements and complex activities remained problematic. Her Y-BOCS scale decreased to 24 over 18 months, and the duration of mealtimes halved.

Further attempts to optimise Ms. A’s residual OCD symptoms were made: the adjuncts risperidone 0.5 mg od, methylphenidate 5 mg od, mirtazapine 7.5 mg od were added cumulatively. These medications offered no benefit with her obsessive-compulsive phenomena. These were discontinued in turn due to worsening extrapyramidal side effects, agitation and social withdrawal, and hypersomnolence respectively. Conservative and non-pharmacologic therapies were also trialled to manage Ms. A’s OCD symptoms to improve her obsessional slowness and break compulsive rituals.

At age 25, Ms. A’s anxiety improved using lorazepam 1 mg bd. Shortly thereafter Ms. A commenced the dopaminergic pramipexole hydrochloride, up-titrated from 0.25 mg to 0.5 mg bd. This significantly improved her obsessional slowness, motor initiation and psychomotor speed, with a reduction in mealtime duration by a further 50%. Further attempts to increase the dose of pramipexole hydrochloride yielded increased initiation and volition, yet increased Ms. A’s anxiety and agitation. She continued with pramipexole hydrochloride 0.5 mg bd. Ms. A had significant functional improvement in undertaking her activities of daily living, increased social engagement and a markedly increased hedonic response.

## Discussion and conclusions

OCD symptoms are a relatively common neuropsychiatric manifestation of SSADH deficiency in adolescents and adults. In their SSADH-deficient patient database (*n* = 33), Knerr et al. [9] found 11 (33%) adolescents and adults displayed OCD symptoms. Similarly, 12 (48%) adults presented with OCD behaviours in Lapalme-Remis’ et al. [10] analysis of their SSADH-deficient database (*n* = 25).

**Fig. 2:**
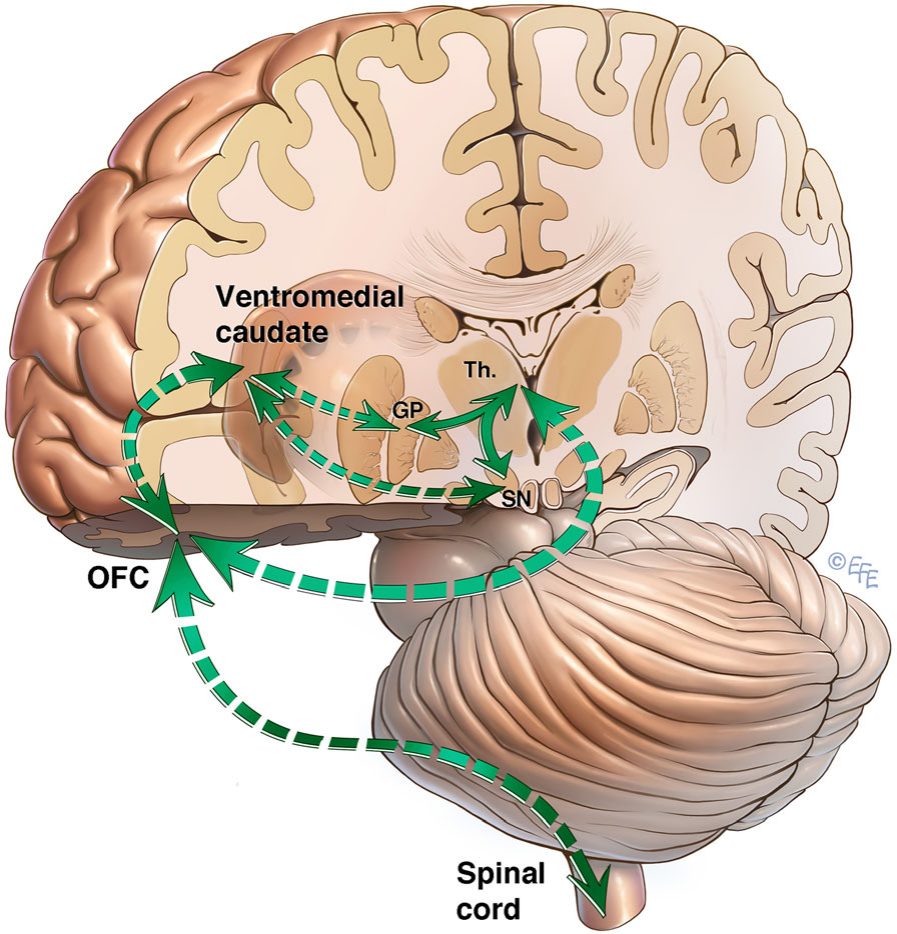
Neural circuit. Lateral orbitofrontal loop, projecting from the orbitofrontal cortex (OFC) via the ventromedial caudate and substantia nigra (SN) to the globus pallidus (GP), then thalamus (Th.), and back to the OFC

Bilateral changes to the globi pallidi have been described in SSADH deficiency, with macroscopic findings including well-circumscribed pallidal discolouration and hyperaemia [13]. These changes correspond with areas of increased T2 signal in the globi pallidi on neuroimaging [13], consistent with the findings reported in this patient. Pallidal pathology could be a possible explanation for this patient’s presentation. The association between pallidal pathology and development of OCD and other compulsive disorders has been well-documented [14–16], with disruption to the integrity of specific frontal-subcortical tracts including the lateral orbitofrontal loop [17] (Fig. 2). Excitatory glutamergic and inhibitory GABAergic cortical tracts from the orbitofrontal cortex and anterior cingulate cortex converge on the globus pallidus via the striatum [18, 19]. Projections from the globus pallidus to the thalamus and then back to the cortex completes this neural circuit [18, 19]. When pathology of the globus pallidus occurs, this results in an imbalance within these neural circuits [19]. This may thus underpin our patient’s constellation of “inhibitory” symptoms (obsessional slowness and hesitancy) and “excitatory” symptoms (motor rituals, stereotypies and agitation). It is possible that the degree of pallidal pathology is associated with OCD symptom severity.

Despite the prevalence of OCD symptoms amongst SSADH deficiency patients, there is a gap in knowledge regarding the optimal management approach. Knerr et al. [9] describe one individual whose anxiety and OCD symptoms responded positively to fluoxetine 20 mg/day used concomitantly with methylphenidate 10–20 mg tds, risperidone 2 mg bd and carbamazepine 20 mg/kg/day. To our knowledge, there is no other literature detailing OCD symptom response to management.

Our case also suggests benefit in using a selective serotonin reuptake inhibitor: Ms. A had moderate improvement in her OCD symptoms using escitalopram. Methylphenidate and risperidone provided no benefit. Use of the stimulant methylphenidate also worsened Ms. A’s comorbid agitation and anxiety. In this regard, we hypothesised an anxiety-based obsessionality underpinned Ms. A’s OCD symptoms.

The dopamine agonist pramipexole hydrochloride was added to address Ms. A’s constellation of OCD and movement symptoms. This is the first report of the utility of this agent in SSADH deficiency. Increasing pramipexole hydrochloride dose increased Ms. A’s motor initiation and psychomotor speed, however, at the cost of increased anxiety and agitation. This reaffirmed our hypothesis of an anxiety-grounding to Ms. A’s obsessive-compulsive phenomena.

OCD symptoms are a possible neuropsychiatric manifestation in individuals with SSADH deficiency. Overall, our case suggests use of the serotonergic agent escitalopram in conjunction with dopaminergic pramipexole hydrochloride is a potentially effective symptomatic treatment regimen in patients with SSADH deficiency presenting with OCD symptoms. Dosage of pramipexole hydrochloride should be titrated according to patient symptoms and side effects. This is particularly important in SSADH-deficient individuals who present with OCD symptoms and anxiety, as their OCD symptoms may have an anxiety-basis. This ensures OCD symptom optimisation whilst preventing increasing agitation. Further studies into the management approach of OCD and other neuropsychiatric symptoms in SSADH deficiency patients are required. The management of this clinical case demonstrates a possible treatment algorithm for SSADH deficiency patients with OCD symptoms, suggesting that serotonergic treatment is the mainstay, but may be augmented by dopaminergic and other therapy to reduce slowness without worsening anxiety or agitation.

## Data Availability

All data generated or analysed during this study are included in this published article.

## Abbreviations

SSADH: Succinic semialdehyde dehydrogenase
OCD: Obsessive-compulsive disorder
GABA: γ-aminobutyric acid
GHB: γ-hydroxybutyric acid
MRI: Magnetic resonance imaging
